# Contrasting Early Ordovician assembly patterns highlight the complex initial stages of the Ordovician Radiation

**DOI:** 10.1038/s41598-022-07822-z

**Published:** 2022-03-09

**Authors:** Farid Saleh, Pauline Guenser, Corentin Gibert, Diego Balseiro, Fernanda Serra, Beatriz G. Waisfeld, Jonathan B. Antcliffe, Allison C. Daley, M. Gabriela Mángano, Luis A. Buatois, Xiaoya Ma, Daniel Vizcaïno, Bertrand Lefebvre

**Affiliations:** 1 Yunnan Key Laboratory for Palaeobiology, Institute of Palaeontology, Yunnan University, Kunming, China; 2 MEC International Joint Laboratory for Palaeobiology and Palaeoenvironment, Institute of Palaeontology, Yunnan University, Kunming, China; 3 Université Claude Bernard Lyon 1, CNRS, UMR5023, LEHNA, Université de Lyon, 69622 Villeurbanne, France; 4 Laboratoire de la Préhistoire à l’Actuel: Culture, Environnement et Anthropologie (PACEA, UMR 5199 CNRS, INEE), University of Bordeaux, Bordeaux, France; 5 Facultad de Ciencias Exactas, Físicas y Naturales, Universidad Nacional de Córdoba, Córdoba, Argentina; 6 Centro de Investigaciones en Ciencias de la Tierra (CICTERRA), Consejo Nacional de Investigaciones Científicas y Técnicas (CONICET), Av. Vélez Sarsfield 1611, CP X5016GCA Córdoba, Argentina; 7 Institute of Earth Sciences, University of Lausanne, Géopolis, 1015 Lausanne, Switzerland; 8 Department of Geological Sciences, University of Saskatchewan, Saskatoon, SK S7N 5E2 Canada; 9 Centre for Ecology and Conservation, University of Exeter, Penryn, UK; 10 Carcassonne, France; 11 Université Claude Bernard Lyon1, École Normale Supérieure de Lyon, CNRS, UMR5276, LGL-TPE, Université de Lyon, Villeurbanne, France

**Keywords:** Macroecology, Palaeoecology, Palaeontology

## Abstract

The Early Ordovician is a key interval for our understanding of the evolution of life on Earth as it lays at the transition between the Cambrian Explosion and the Ordovician Radiation and because the fossil record of the late Cambrian is scarce. In this study, assembly processes of Early Ordovician trilobite and echinoderm communities from the Central Anti-Atlas (Morocco), the Montagne Noire (France), and the Cordillera Oriental (Argentina) are explored. The results show that dispersal increased diachronically in trilobite communities during the Early Ordovician. Dispersal did not increase for echinoderms. Dispersal was most probably proximally triggered by the planktic revolution, the fall in seawater temperatures, changes in oceanic circulation, with an overall control by tectonic frameworks and phylogenetic constraints. The diachronous increase in dispersal within trilobite communities in the Early Ordovician highlights the complexity of ecosystem structuring during the early stages of the Ordovician Radiation. As Early Ordovician regional dispersal was followed by well-documented continental dispersal in the Middle/Late Ordovician, it is possible to consider that alongside a global increase in taxonomic richness, the Ordovician Radiation is also characterized by a gradual increase in dispersal.

## Introduction

The fossil record of the Cambrian Period documents the earliest evolution and diversification of eumetazoans on Earth^1^, during which animals developed a wide range of morphological features and characteristics^2,3^. The ancient ecology of these animals can be reconstructed based on the exceptional fidelity of anatomy preserved in Burgess Shale (BS)-type fossil sites^4^, such as Burgess Shale, Chengjiang, and Qingjiang biotas^4–6^. The Cambrian Explosion was followed by the Ordovician Radiation^2^. During the Ordovician, diversity significantly increased and was accompanied by structural changes in ecosystems^7–9^. However, most palaeoecological studies have focused on the Middle Ordovician and onwards. As a result, the Early Ordovician remains an understudied interval, despite having the potential to help elucidate the transition between the Cambrian Explosion and the Ordovician Radiation, particularly given that the fossil record of the late Cambrian is scarce (i.e., the Furongian gap)^10^.

**Figure 1:**
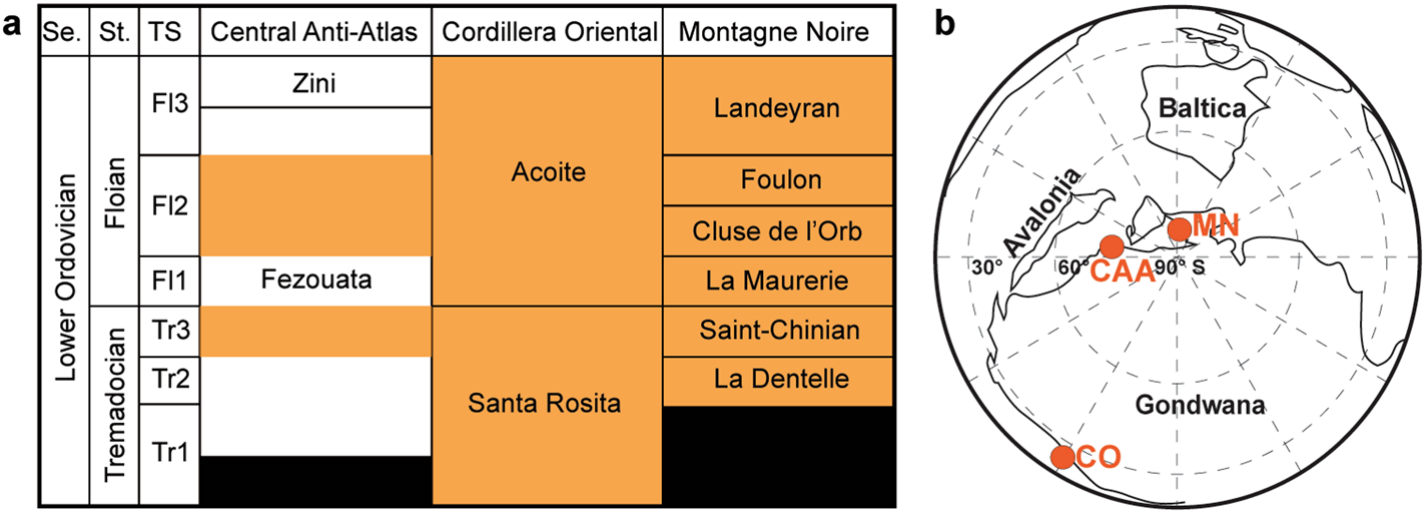
(**a**) Stratigraphic correlations between the Central Anti-Atlas (CAA), the Montagne Noire (MN), and the Cordillera Oriental (CO). The studied intervals within this work are highlighted in orange. Note that for the Fezouata Shale orange intervals were not selectively chosen, as those are the only ones yielding echinoderm and trilobite fossils. Se. = Series, St. = Stage, TS = Time Slices^27^; Morocco^28^; Cordillera Oriental^19^; Montagne Noire^14^. (**b**) Paleogeography of the three regions; modified from Saleh et al.^23^.

**Figure 2:**
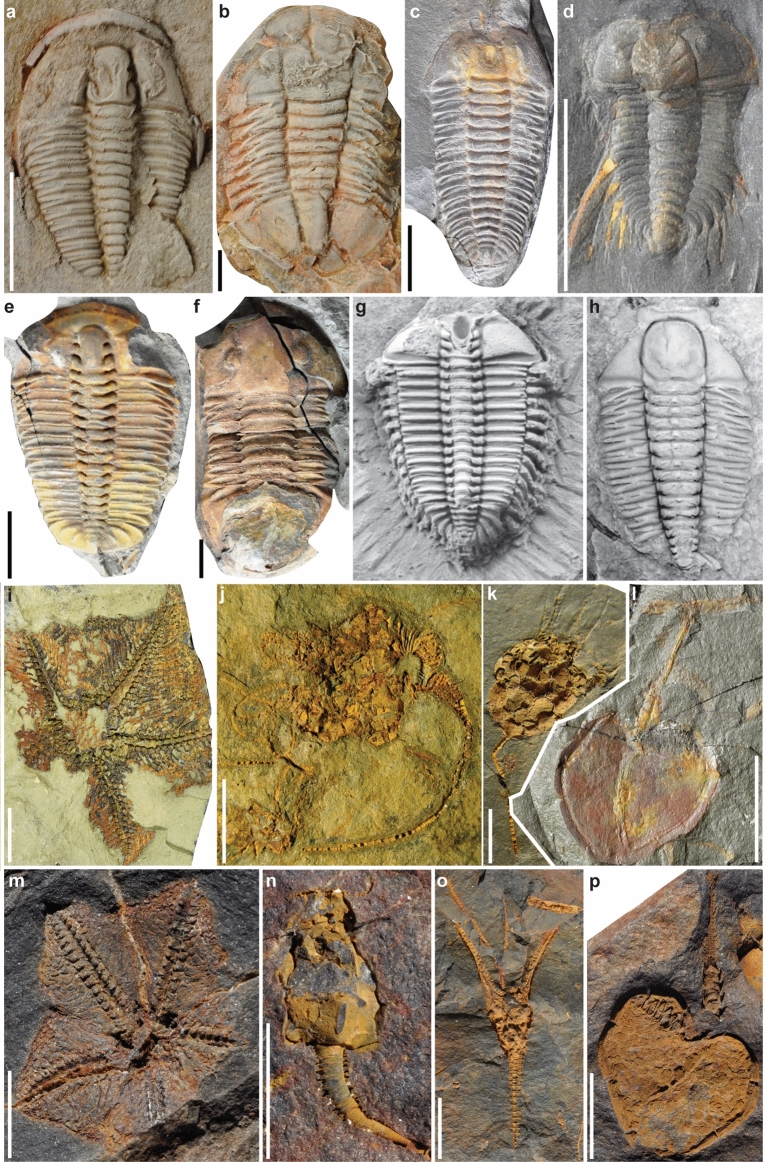
Early Ordovician trilobites from the Central Anti-Atlas (**a**–**d**), the Montagne Noire (**e**,**f**), and the Cordillera Oritental (**g**,**h**) in addition to Early Ordovician echinoderms from the Central Anti-Atlas (**i**–**l**), and the Montagne Noire (**m**–**p**). (**a**) *Euloma* sp., late Tremadocian; AA.TGR1c.OI.232, (**b**) *Platypeltoides magrebiensis*, late Tremadocian; AA.TGR0a.OI.132, (**c**) *Bavarilla zemmourensis*, late Tremadocian; AA.BIZ15.OI.16, (**d**) *Anacheirurus adserai*, late Tremadocian; AA.BIZ15.OI.291, (**e**) *Euloma filacovi*, late Tremadocian; UCBL-FSL 740109, (**f**) *Paramegalaspis immarginata*, late Tremadocian; UCBL-FSL 740027, (**g**) *Pliomeridius sulcatus,* middle Floian; CEGH-UNC-16311, (**h**) *Leptoplastides granulosa*; middle Tremadocian, CEGH-UNC-21324, (**i**) *Villebrunaster* sp., late Tremadocian; FSL 424 961, (**j**) *Macrocystella bohemica*, late Tremadocian, MHNM.15690.196, (**k**) Eocrinoidea indet., late Tremadocian; MHNM.15690.141, (**l**) Cornuta n. gen. n. sp. cf. "*Phyllocystis*" *jingxiensis*; FSL.712971, (**m**), *Villebrunaster thorali*, late Tremadocian; UCBL-FSL 168673, (**n**) *Macrocystella azaisi*, late Tremadocian; MBB-GG50, (**o**) *Aethocrinus moorei*, late Tremadocian; MNHN.A49684, (**p**) *Phyllocystis blayaci*, late Tremadocian; UCBL-FSL 168703. 1 cm scale bars (**a**–**f**; **i**–**p**), magnification = 0.8× (**g**) and 1.8× (**h**). Abbreviations (repositories): *AA* Cadi-Ayyad University, Marrakesh (Morocco), *MBB* Musée du Biterrois, Béziers (France), *MNHN* Muséum National d'Histoire Naturelle, Paris (France), *UCBL-FSL* palaeontological collections, Lyon 1 University, Villeurbanne (France), *CEGH-UNC* Universidad Nacional de Córdoba, Córdoba (Argentina).

**Figure 3:**
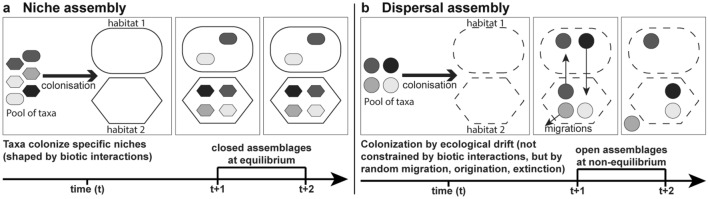
Niche- versus Dispersal-assembly perspectives. (**a**) In a niche-assembly scenario, communities are closed and balanced, built by a limited number of species at equilibrium (similar assemblages at t + 1 and t + 2). Niche taxonomic composition is controlled by deterministic (i.e., non-random) biotic interactions between taxa and their environment. (**b**) Dispersal-assembly scenario in which communities are constantly changing (i.e., non-equilibrium; different assemblages between t + 1 and t + 2) owing to random processes such as migrations.

This work investigates the ecological processes that structure community composition during the Early Ordovician of three regions: The Central Anti-Atlas (CAA; Morocco), Montagne Noire (MN; France), and Cordillera Oriental (CO; Argentina) (Fig. 1a,b). Because not all regions preserve all types of taxa owing to taphonomic biases^11,12^, this study is focusing on echinoderms and trilobites as these are readily preserved and are well studied in the three regions (Fig. 2)^13–23^. Moreover, trilobites are the most abundant and diverse group in the three studied regions followed by echinoderms (in the CAA and the MN)^13–23^. Communities can be niche-assembled (Fig. 3a) when they are at equilibrium, closed, and balanced, with a stable taxonomic composition maintained by deterministic processes like positive (e.g., mutualism) and negative (e.g., competition) interactions between individuals^24–26^. Communities are dispersal-assembled (Fig. 3b) when they are open, in non-equilibrium state with their compositions depending on continuous dispersion between communities, and subjected to random originations, and extinctions^24–26^. Once seen as incompatible hypotheses, niche- and dispersal-perspectives are now seen as opposite ends of a continuum^24–26^. Within this continuum, the taxonomic composition of an assemblage may result to a greater or lesser extent of each process, or from an equal combination of the two^24–26^.

Identifying niche and dispersal processes is a fundamental question in modern ecology and has become a central theme for conservation studies^24^. Novel approaches have been developed that can identify assembly-processes within fossil datasets, and describe changes in processes over evolutionary timescales, and their potential links with abiotic and biotic events^24^. In order to quantify niche and dispersal processes within trilobite and echinoderm assemblages in the three regions, this work applies the novel Dispersal and Niche Continuum index (DNCI) analyses to understand changes in community assembly between the Tremadocian and the Floian^24^, i.e., the two Early Ordovician stratigraphic stages. The DNCI quantifies the relative contribution of niche and dispersal processes to the taxonomic dissimilarity between assemblages, which are defined as statistical clusters of fossiliferous levels in each region^24^. The DNCI ranges from significantly positive (i.e., niche-assembled community) to significantly negative (i.e., dispersal-assembled community) values. The lower the value of the DNCI, the more important dispersal was in a certain assemblage^24^. A community was equally niche- and dispersal-assembled when the mean DNCI and its confidence interval overlap with zero^24^. Once niche and dispersal contributions were quantified, they are then placed in the larger evolutionary framework of trilobites and echinoderms in addition to the geological context of the three localities.

From a terminological perspective, the generic term “assemblage” is used to refer to all taxa inhabiting a particular area, regardless of the spatial scale considered (e.g., community, meta-community, bioregions). “Assembly-processes” (i.e., niche and dispersal) are the ecological forces bringing together and maintaining these taxa within assemblages. Niche- and dispersal-assembly should be differentiated as terms from cosmopolitanism and endemism. For instance, within a community model, if dispersal is the only ecological force considered, this can lead to an endemic and/or a cosmopolitan signal.

## Results and discussion

**Figure 4:**
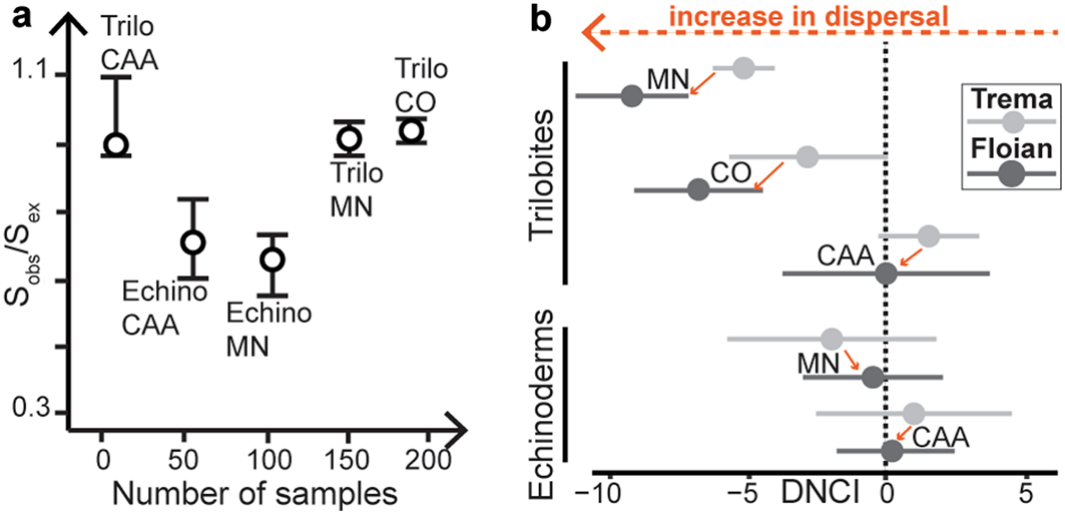
(**a**) Estimation of the sampling effort [observed sampling (S_obs_) over extrapolated sampling (S_ex_)] of echinoderms (echino) and trilobites (trilo) in the Central Anti-Atlas (CAA), the Montagne Noire (MN), and the Cordillera Oriental (CO). S_obs_ and S_ex_ were also calculated while separating between the Floian and the Tremadocian of the three regions (Supplementary Material 1). (**b**) DNCI analyses for trilobites and echinoderms during the Tremadocian (Trema) and Floian of the Central Anti-Atlas, the Montagne Noire, and the Cordillera Oriental.

Trilobites in the CAA are assembled equally by both niche- and dispersal-processes during the Early Ordovician (i.e., the range of the DNCI and its confidence interval overlap with zero in the Tremadocian and the Floian; Fig. 4b). Trilobites in the MN are dispersal-assembled in the Tremadocian and the Floian (i.e., DNCI is negative and does not overlap with zero; Fig. 4b). Trilobite assemblages in the CO are equally driven by both niche and dispersal processes in the Tremadocian and mainly driven by dispersal processes in the Floian (Fig. 4b). Trilobites in the MN and CO show little overlap between the Tremadocian and the Floian (Fig. 4b). This indicates a clear difference between the Tremadocian and the Floian records of trilobites in the CO and the MN. This difference is characterized by a transition towards more negative DNCI values (Fig. 4b) associated with a decrease in niche processes, and an increase in dispersal^24^. The contrasting results for trilobites between the three regions (Fig. 4b) do not reflect a differential sampling effort bias because trilobites in the CAA, the CO, and the MN are equally sampled (i.e., S_obs_/S_ex_ ~ 0.9; Fig. 4a).

The increase in trilobite regional dispersal during the Early Ordovician (at least in the MN and the CO) correlates with environmental changes during this time interval. During the Furongian, the planktic revolution started, and increased the availability of a globally distributed food source for animals^29^. Furthermore, temperatures achieved a global hothouse at the onset of the Furongian (also known as the Steptoean Positive Carbon Isotope Excursion or SPICE event) and started to decrease right afterward in the early/middle Furongian up until the Late Ordovician^30^. The global cooling trend established a more prominent global oceanic circulation in the Middle/Late Ordovician than in the late Cambrian^31,32^. This means that at least transitional conditions of oceanic ventilation and circulation existed in the Tremadocian and the Floian (periods of high sea level)^33^. Thus, it is most likely that oceanic circulation, cooling, and the planktic revolution facilitated dispersal because these environmental changes are linked to animals’ specific evolutionary patterns and ecological behavior^29^. For instance, an increased colonization of deep-sea floors is indicated by ichnologic evidence, and took place during the Early Ordovician^34^. This increased animal activity in deep seas has been attributed to augmented oceanic ventilation^34^. Moreover, in the Furongian, for the first time, trilobites started to disperse into new habitats by exploring brackish water environments, a trend that continued well into the Middle Ordovician^20,35^. Additionally, most of the known early to middle Cambrian trilobite larvae were not spherical but flat in shape (adult-like protaspides) and are interpreted as having lived on or near the seafloor^36–39^. In the Furongian, some trilobite groups developed bulbous configurations^36^. Bulbous larvae (non-adult-like protaspides) were adapted to a planktic mode of life^40^. Although bulbous larvae have so far been found in few Ordovician lineages^36,40^, Bignon et al.^41^ argued that this type of larvae must have been more abundant. Thus, some of the lineages yielding either bulbous larvae or juveniles of this type might explain the observed increase in trilobite dispersal. However, this evolutionary trait only explains the increase in dispersal but it does not explain why trilobite dispersal was non-uniform between regions. The non-uniform dispersal pattern might be resulting from the limited temporal sampling in the CAA (Fig. 1a), which represents a significantly more restricted window in comparison to the CO, and the MN (Fig. 1a). It can also result from differences in regional settings (e.g., geodynamic framework). During the Early Ordovician, the CAA was a restricted area^42,43^. As such, the CAA was isolated from the global oceanic circulation (i.e., the newly established global currents did not increase inter-communication between CAA sites), which may explain why trilobites did not disperse within this area as early as trilobites in other regions (Fig. 4b). Moreover, the CO was partially protected by a volcanic arc to the west which might explain why the composition of the assemblages of this area remained equally niche- and dispersal-assembled in the Tremadocian in comparison to the dispersal-assembled MN^44^.

Another aspect of these data is that echinoderms in the CAA and the MN are assembled equally by both niche- and dispersal-processes during the Early Ordovician, without showing a change in community assembly processes (Fig. 4b). Due to sampling biases, trilobite and echinoderm data cannot be directly compared [i.e., S_obs_/S_ex_(echinoderm) ~ 0.6 < S_obs_/S_ex_(trilobite) ~ 0.9; Fig. 4a]. However, the similarity between echinoderms in the CAA and the MN is not biased by sampling (i.e., S_obs_/S_ex_ ~ 0.6 for both regions; Fig. 4a). This essentially means that echinoderm assembly patterns were uniform between regions and were not differentially affected by the environmental changes happening at the global scale. Unfortunately, it is impossible to further discuss this finding owing to the absence of fossilized echinoderm larvae. It is assumed that the possession of planktotrophic larvae is a plesiomorphic condition in the five extant classes^45^, which appeared and diversified either in the Early Ordovician (asteroids, crinoids, ophiuroids) or in the Darriwilian (echinoids, holothuroids)^46^. However, confirming that echinoderm planktotrophic larvae were acquired by the Early Ordovician, is not possible^47^.

The relevance of dispersal on assemblages is also dependent on the possibility for new immigrants to enter local communities (i.e., openness of communities). Therefore, an increase in dispersal in community assembly, also suggests that local communities became more susceptible to invasion by immigrants. Such a change can be driven by an increase in the amount of unused resources^48^ related to the diversification of phytoplankton during the plankton revolution^29^. The increase in dispersal could also result from transient taxa^49^ as new dispersive morphologies (e.g., bulbous larvae) enable the development of new demographics (i.e., source/sink)^50^ and evolutionary (increased taxa longevity) dynamics^51^. For instance, non-specialized taxa could survive in remote and hostile environments if they possessed an adequate dispersive morphology that allowed for a continuous influx of new individuals from their source community (i.e., competition-colonization trade-off).

With these new data, it is now possible to place the transition between the Cambrian Explosion and the Ordovician Radiation in an ecological context. The Cambrian Explosion is primarily linked with increased niche packing and partitioning^52^. Previous studies made at a global scale suggested that niche-processes remained high during the first two Ordovician stages^52^, indicating that the remarkable decrease in niche-assembly was only acquired later, starting in the Middle Ordovician^53^. The overlap of the DNCI with zero in the Tremadocian, and to some extent in the Floian, indicates that both niche and dispersive regimes are equally detected in the analyses, highlighting that the Early Ordovician was a time of ecological transition between the Cambrian Explosion and the Ordovician Radiation. This study provides a new perspective, complementing previously described dynamics by showing that changes in community assembly (i.e., increase in dispersal in comparison to the Cambrian) can already be observed in the Early Ordovician, especially at a regional scale.

## Conclusion

The Early Ordovician is a complex and poorly understood time in the evolution of life on Earth. Dispersal increased during this time and this was likely triggered by evolutionary dynamics (e.g., the planktonic revolution) with underlying environmental forcing (e.g., oceanic cooling and circulation changes). However, dispersal increase was not homogenous for all animal groups and regions (Fig. 4b) as it was controlled by a combination of clade inherent features and the tectonic framework of each region. The importance of the results presented herein is that they (1) were obtained using a novel method that is independent of classical taxonomic richness metrics, and (2) highlight the complexity of the early stages of the Ordovician Radiation. Considering that diachronous regional dispersal in the Early Ordovician was followed by episodes of continental dispersal during the Middle Ordovician^54–57^, it is possible to suggest that the Ordovician Radiation can be envisaged as facilitated by a set of diachronous (i.e., in one region prior to another) increases (i.e., from regional to continental) in dispersal. This increase in dispersal could also represent a transition from fragmented habitat patches separated by non-livable oxygen-depleted zones in the Early Ordovician to a more homogenous ocean that is dominantly cooler and oxygen-rich in the Middle Ordovician^58^. In the future, it is essential to apply DNCI analyses to a wider range of regions in order to investigate whether either latitudinal or longitudinal dispersal gradients exist during this time interval. These future studies will bring insights on the spreading of regional dispersal during the Early Ordovician, and on how to calibrate the diachronicity of dispersal during this critical time interval.

## Materials and methods

### Studied fossils and regions

The echinoderm and trilobite data in this study are primarily field-based and result from intensive sampling in the three investigated areas over several decades. Data from the Montagne Noire are based on the public collections of Lyon 1 University, France. The Central Anti-Atlas data was extracted from the public collections of Cadi Ayyad University, Morocco, while data from the Cordillera Oriental is from both the literature and from the public collections at the National University of Córdoba, Argentina. This work comprises more than 300 well-dated levels (Fig. 1a). “Levels” represent spatial–temporal limited intervals. For instance, two stratigraphically distinct fossiliferous horizons occurring at the same site were considered as two different levels. Moreover, two coeval fossiliferous layers were considered as distinct "levels", when they occur in two different sites of a same region. Additional detailed information on the studied fossils is provided in Supplementary Material 1 (SM1).

In order to avoid local depositional biases on the analyses, occurrence data within the same range of depositional conditions between the fair-weather wave base and the storm-weather wave base (i.e., offshore) were included in this study. Moreover, there is no dominant oxygen or salinity stress during deposition of most investigated levels and the three regions have a very comparable substrate^19,22,59^. Therefore, levels in which an oxygen stress is evident in the CAA^22^ were excluded from the analyses. During the Early Ordovician, the CAA was a relatively restricted sea^42,43^. The CO was partially protected by a volcanic arc to the west^44^. The MN was an open sea in direct contact with the ocean^42,43^. More information on the geological context is provided in the expanded materials and methods section in SM1.

### Statistical and ecological approaches

Incidence matrices reflecting occurrences (absence/presence) in all layers for each region were built (i.e., five datasets; Supplementary Material 2). The sampling effort observed as a ratio between observed and extrapolated sampling (S_obs_/S_ex_) was calculated using the iNEXT package on R software^60^ and its confidence interval was obtained using the function “Ratio of counts CI” of the software PAST^61^ and is reported on Fig. 4a. Each dataset (e.g., echinoderms in the CAA) was then split into two subsets (e.g., Tremadocian and Floian echinoderms in the CAA; Supplementary Material 3). Cluster analyses (UPGMA) were performed on each subset, and results are present in SM3. The significance of these clusters was then tested and confirmed as statistically significant with an analysis of similarity (i.e., ANOSIM; SM3). These analyses were performed in PAST using the Jaccard index, which is the most commonly used index in paleoecological investigations^62^. DNCI identifies the main assembly process driving species composition between clusters by comparing empirical and null SIMPER profiles^63^. The SIMPER method has been designed to compute and depict the contribution of taxa to the overall taxonomic dissimilarity (OAD) between two clusters/groups of assemblages/communities^63^. The contribution of taxa to OAD depends on the relationship of β-diversity within clusters of assemblages and β-diversity between clusters of assemblages (Supplementary Material 1). In other words, SIMPER considers: (1) taxa that are largely distributed within one cluster, (2) taxa that are largely and evenly distributed between two clusters; (3), rare taxa that are evenly distributed and (4) rare taxa that are only distributed within one cluster. Then an empirical SIMPER profile is produced by ordering the contribution of each taxa to the OAD. This profile is then compared to three null permuted SIMPER (PERSIMPER) profiles (only niche, only dispersal or the equal contribution of niche and dispersal processes). The closer the null profile, the more important is the respective assembly model (Supplementary Material 1). In this sense, the taxonomic dissimilarity and the β-diversity are at the core of PER-SIMPER/DNCI. However, there is no correlation between dissimilarity indices or β-diversity variations and DNCI variations. To perform DNCI analyses, sheets in *SM3* (i.e., 10 in total) were exported as CSV files and are present in a compressed folder (Supplementary Material 4). Three levels that do not belong to any cluster (i.e., highlighted in yellow in SM3) were removed from SM4 because a cluster has to have by definition more than one level to be used in the following DNCI analyses^24^. Clusters also need to be composed of a similar number of levels in order to avoid bias on niche and dispersal processes identification^63^. If the compared clusters are uneven, DNCI will shift toward zero, leading to a false identification of an equal contribution of niche and dispersal processes^24^. A subsampling procedure can be used to make the clusters of equal size and correct this bias. In this case, when clusters differ in size (e.g., cluster 1 has 40% more levels than cluster 2), a subsampling randomly reducing the larger cluster(s) to the size of the smallest cluster was implemented 100 times to minimize the sampling bias effect and produce robust mean DNCI values. DNCI analyses were done using DNCImper^64^ R package available on Github (https://github.com/Corentin-Gibert-Paleontology/DNCImper). A customized R Markdown script of DNCI analyses is provided in Supplementary Material 5. More details regarding the methods used, the choices made, in addition to explanations on how the DNCI operates are provided in SM1.

## Supplementary Information

Supplementary Information 1.

Supplementary Information 2.

Supplementary Information 3.

Supplementary Information 4.

Supplementary Information 5.

Supplementary Legends.

## Data Availability

All data needed to evaluate the conclusions in the paper are present in the Supplementary Materials. Additional data related to this paper may be requested from the authors.
