# Supplementary material for: Contrasting Early Ordovician assembly patterns highlight the complex initial stages of the Ordovician Radiation
Source: Sci Rep. 2022 Mar 9;12:3852. doi: 10.1038/s41598-022-07822-z (PMC8907272; doi:10.1038/s41598-022-07822-z)
Supplement: Supplementary file 1 — Supplementary Information 1. [file 41598_2022_7822_MOESM1_ESM.docx]

SUPPLEMENTARY MATERIAL 1

Additional geological context, material and methods, and results for:

Contrasting Early Ordovician assembly patterns highlight the complex initial stages of the Ordovician Radiation

Farid Saleh,^1,2*^ Pauline Guenser,^3^ Corentin Gibert,^4*^ Diego Balseiro,^5,6^ Fernanda Serra^5,6^ Beatriz G. Waisfeld,^5,6^ Jonathan B. Antcliffe,^7^ Allison C. Daley,^7^ M. Gabriela Mángano,^8^ Luis A. Buatois,^8^ Xiaoya Ma,^1,2,9*^ Daniel Vizcaïno,^10^ Bertrand Lefebvre^11^

^1^Yunnan Key Laboratory for Palaeobiology, Institute of Palaeontology, Yunnan University, Kunming, China

^2^MEC International Joint Laboratory for Palaeobiology and Palaeoenvironment, Institute of Palaeontology, Yunnan University, Kunming, China

^3^Université de Lyon, Université Claude Bernard Lyon 1, CNRS, UMR5023, LEHNA, 69622 Villeurbanne, France.

^4^Laboratoire de la préhistoire à l’actuel: culture, environnement et anthropologie (PACEA, UMR 5199 CNRS, INEE), University of Bordeaux, Bordeaux, France.

^5^Facultad de Ciencias Exactas, Físicas y Naturales, Universidad Nacional de Córdoba, Córdoba, Argentina

^6^Consejo Nacional de Investigaciones Científicas y Técnicas (CONICET), Centro de Investigaciones en Ciencias de la Tierra (CICTERRA), Av. Vélez Sarsfield 1611, CP X5016GCA, Córdoba, Argentina

^7^Institute of Earth Sciences, University of Lausanne, Géopolis, CH-1015 Lausanne, Switzerland

^8^Department of Geological Sciences, University of Saskatchewan, Saskatoon SK S7N 5E2, Canada

^9^Centre for Ecology and Conservation, University of Exeter, Penryn, UK

^10^ 7 rue Chardin, Maquens, F-11090 Carcassonne, France

^11^Université de Lyon, Université Claude Bernard Lyon1, École Normale Supérieure de Lyon, CNRS, UMR5276, LGL-TPE, Villeurbanne, France

*corresponding authors: F. Saleh ([farid.nassim.saleh@gmail.com](mailto:farid.nassim.saleh@gmail.com)), C. Gibert (corentingibert@gmail.com), X. Ma ([X.Ma2@exeter.ac.uk](mailto:X.Ma2@exeter.ac.uk))

**Collection and Field Information**

Data from the Montagne Noire are based on the public collections of Lyon 1 University, France, which is the most exhaustive collection of Lower Ordovician faunas from this ~1,250 km^2^ area (Minervois and Pardhaillan nappes). Resulting from over 40 years of sampling, this collection comprises 5,213 specimens (rock samples) containing over 8,000 distinct fossils belonging to more than 200 different taxa (annelids, arthropods, brachiopods, bryozoans, echinoderms, hemichordates, molluscs, scyphozoans, and sponges) [1]. The precise stratigraphic position (horizon) and geographic origin (locality) of each sample are well documented [1-4]. In the Montagne Noire, the Lower Ordovician corresponds to a ~2,000 m thick sequence represented by the La Dentelle, Saint-Chinian, La Maurerie, La Cluse de l'Orb, Le Foulon, and Landeyran formations [1-6].

The collections of the Cadi Ayyad University, Marrakesh provide a relatively similar field-based dataset for a well-delimited, about 900 km^2^ wide area within the Central Anti-Atlas (Ternata plain, Zagora area). The Marrakesh collections were built in the last 20 years through successive campaigns of sampling and detailed logging of the ~900 m thick Lower Ordovician Fezouata Shale in the CAA area [7]. These collections include 5,289 specimens (rock samples) preserving over 8,000 fossils representing around 180 taxa (annelids, arthropods, brachiopods, echinoderms, hemichordates, molluscs, scyphozoans and sponges) [8]. The precise stratigraphic position (fossiliferous layer) is well known for each specimen [9-12].

Data from the Cordillera Oriental of Northwest Argentina derive from intensive field collection of trilobites through the last three decades (housed at the Centro de Investigaciones Paleobiológicas collection, at the University of Córdoba) and data compiled from the literature. The dataset includes 524 samples, from an area of about 7000 km^2^ for Tremadocian localities and about 3500 km^2^ for Floian ones. Trilobite occurrences are referred to a refined stratigraphic framework [13-15] and are biostratigraphically well constrained based on graptolite, conodont, and trilobite faunas [16-18]. Tremadocian and Floian deposits are included in the Santa Rosita and Acoite formations (and coeval units). Integrated thickness from Tremadocian successions from eastern localities of the Cordillera Oriental attains ~1900 m, whereas Floian successions reach 2300 m in the western side of the basin.

Siliciclastic facies in the three regions are generally stacked forming coarsening-upward parasequences, representing progradation of adjacent belts of wave-dominated shallow-marine sub-environments that may encompass from the shelf (i.e., below storm wave base) to the foreshore (i.e., between the high and low tide lines). Most commonly, however, shoreface and offshore belts are represented [e.g., 14]. Rapid facies shifts are typically restricted to flooding surfaces bounding the parasequences. Taxa from the three regions lived under a similar bathymetry and comparable hydrodynamic energy conditions [1, 19, 20]. Moreover, there is no dominant oxygen or salinity stress during deposition of most investigated levels and the three regions have a very comparable substrates in terms of grain size (alternation of sandstone and mudstone) and degree of consolidation (softgrounds) [1, 19-22]. Sedimentation rates in the three areas are broadly comparable, essentially reflecting the alternation of slow fallout deposition during fair-weather times and rapid deposition during storm events, the latter associated with variable levels of erosional reworking [22]. Based on these environmental criteria, more than 300 levels were retained for the analysis.

**Scale of Analyses**

The decision to divide the database into Tremadocian and Floian subsets was made because numerous previous studies were able to qualitatively assess a change in community diversity and structure at this scale [3-6, 9-18]. It is worth noting that we did not opt for a finer scale consisting of dividing the original dataset into Tr1, Tr2, Tr3, Fl1, Fl2, and Fl3, because results would be fragmentary as fossils from the CAA that are used in this study were found either in the Tr3, or the Fl2 [9-12]. This temporally limited distribution of fossils in the CAA does not cause a problem because even when the Tremadocian and Floian are not fully preserved, we would still be investigating comparable time intervals (Tr3 *vs*. Fl2). Thus, for both practical and paleontological reasons we argue that the most adequate scale to choose for following analyses is the one dealing with the Tremadocian and the Floian as separate subsets.

**Jaccard Index Usage**

The Jaccard index is chosen because it is the easiest way to represent the degree of similarity between two assemblages. It considers the shared and not shared taxa equally. Its value corresponds to the ratio of common taxa related to the taxa that are not shared, which allows visualizing raw similarity between two assemblages. It is also the widest used similarity index in palaeontological studies on presence/absence data.

**Floian-Tremadocian Sampling Effort**


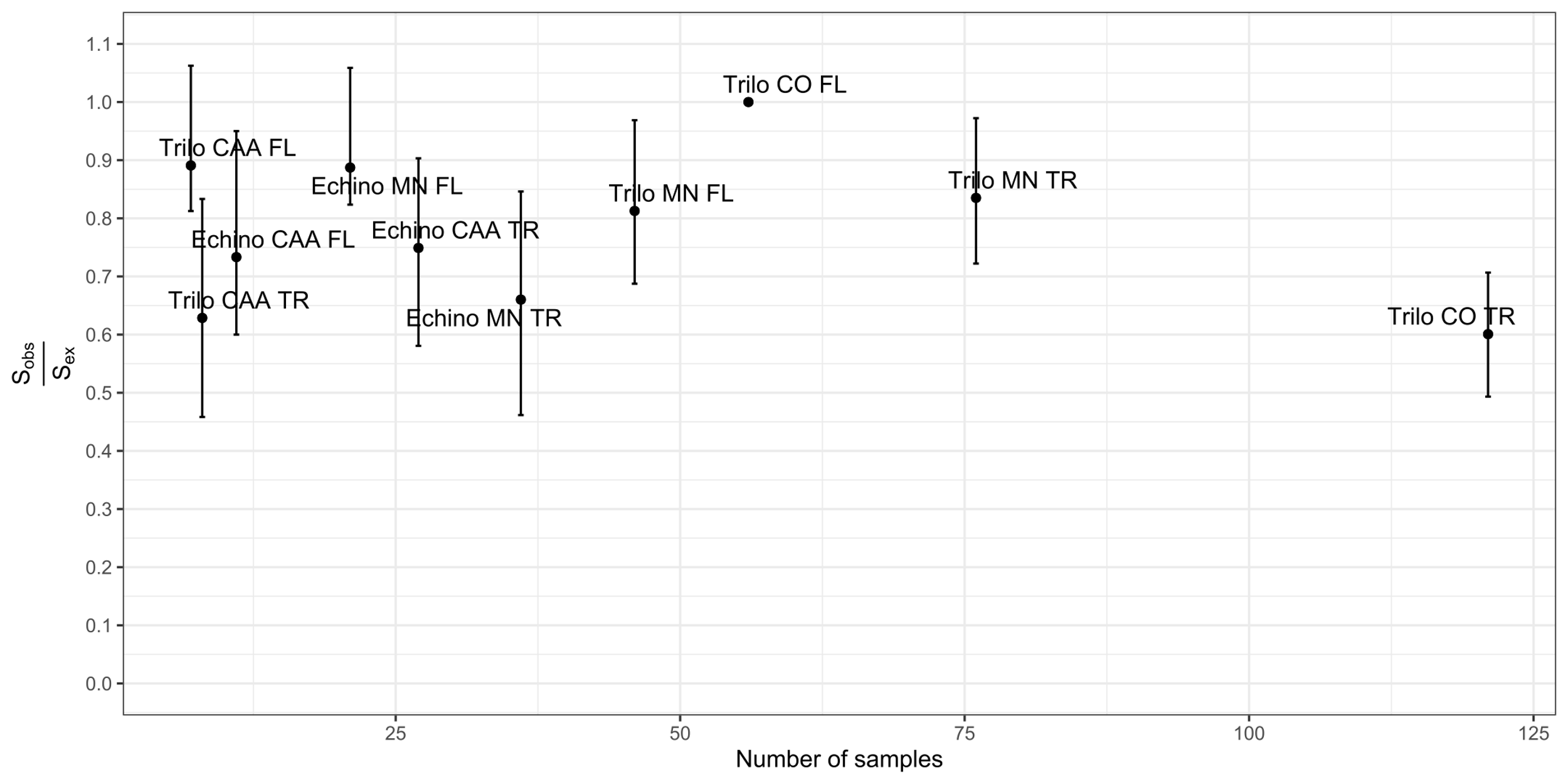


**Supplementary Figure 1.** Results of the sampling effort while separating Floian and Tremadocian data. Sampling is comaprable between most groups. The only exception is in the CO, where Tremadocian trilobites are less-well samples than Floian ones. However, the increase in dispersal observed in this region is unlikely to be resulting from this sampling effort bias, because Serra et al., 2019 [19], obtained a similar conclusion (increase in dispersal), based on a completely different method.

**Further DNCI information**

DNCI is a quantitative index based on the SIMPER (SIMiliarity PERcentage) and PER-SIMPER (PERmutations of a SIMPER) qualitative approaches for inferring community assembly processes (Fig. S2). These methods require taxon/region occurrence dataset contrary to most methods used in modern ecology based on complex information that are often unreachable in paleontological datasets (e.g., abundance, environmental matrix, highly resolved phylogeny) [23]. DNCI needs as well to be computed with significantly dissimilar clusters because the original SIMPER method is made to estimate the contributions of taxa (in percentage) to the overall dissimilarity between two or more clusters [24]. PER-SIMPER original method is based on permutating the SIMPER matrix (i.e., an occurrence matrix divided in two or more groups/clusters) under three conditions corresponding to niche, dispersal or niche+dispersal hypotheses with fixed row sums (i.e., taxonomic richness of levels), fixed column sums (i.e., number of levels where a species is found) and fixed row+column sum permutations, respectively. The null PER-SIMPER profiles generated by the three permutations are then compared with the empirical SIMPER profile: the closer to the empirical profile, the more important is the respective assembly process in the analyzed assemblage (Fig. S2). The intensity of the deviation between empirical and permuted profiles is estimated with three E indexes (i.e., logarithm of the sum of squared deviations between two profiles). These indexes are then used to qualitatively identify the main assembly process in PER-SIMPER. Based on the three E-metric values, the DNCI can quantitatively estimate the intensity of dispersal versus niche processes. DNCI analyses were done using DNCImper [25] R package available on Github (<https://github.com/Corentin-Gibert-Paleontology/DNCImper>). A customized R Markdown script of DNCI analyses is provided in the *Supplementary Material 4*.

**
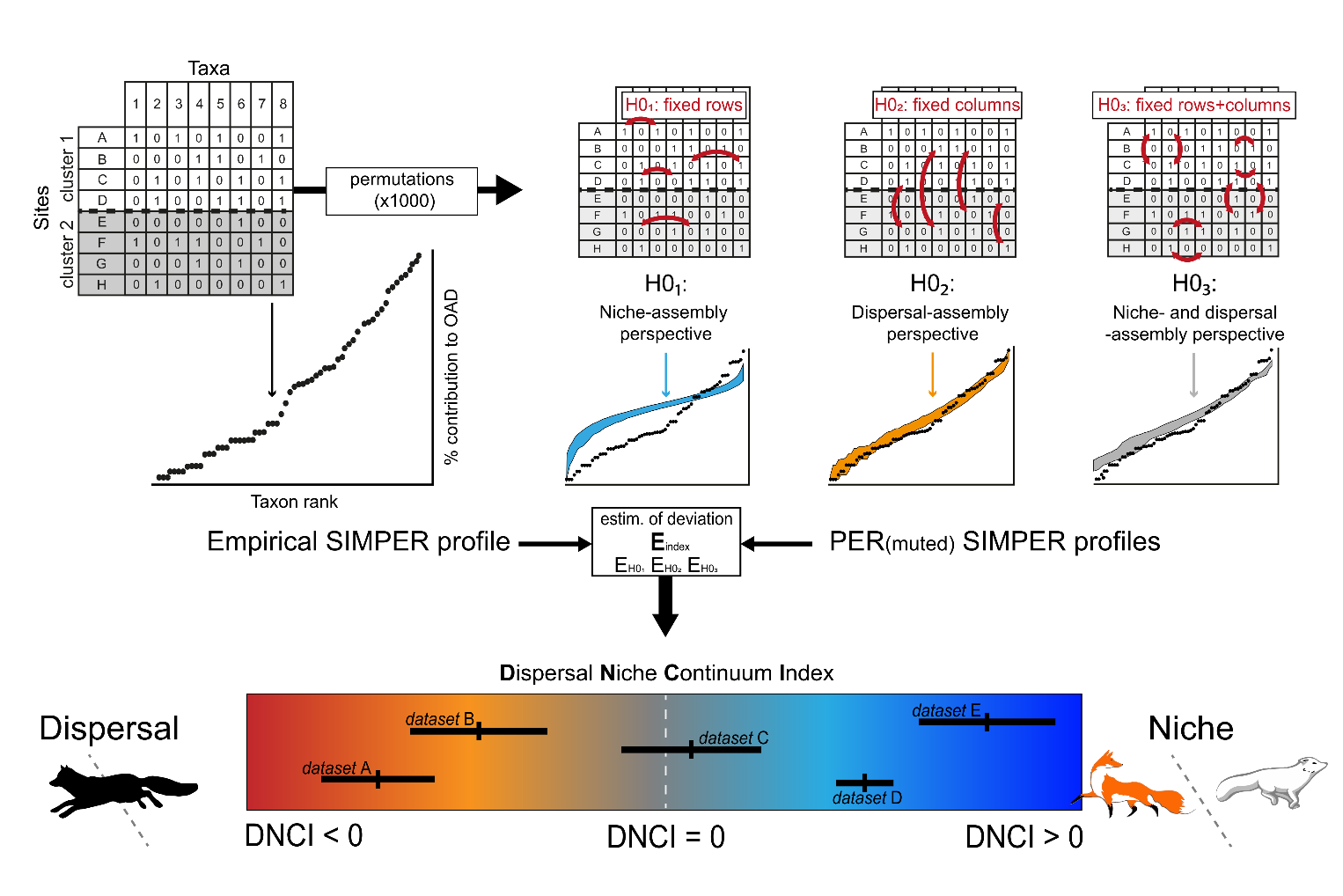
Supplementary Figure 2.** Diagram of the permutation method (PER-SIMPER) and the Dispersal Niche Continuum Index (DNCI) computation. The empirical matrix (top-left corner) is permuted under three conditions to produce three null PER-SIMPER profiles corresponding to the three null hypotheses of assembly (H0_1_, H0_2_, H0_3_). E index is then used to estimate the deviation between the empirical and permuted profiles. The three E indexes (EH01, EH02, EH03) corresponding to the deviation of the three-null model with the empirical profile are used to compute DNCI; from Gibert et al., [26].

**REFERENCES**

1. Vizcaïno, D., Álvaro, J.J. and Lefebvre, B., 2001. The lower Ordovician of the southern Montagne Noire. Annales de la Société géologique du Nord, 8(4), pp.213-220.
2. Vizcaïno, D. and Álvaro, J.J., 2002. Adequacy of the Early Ordovician trilobite record in the southern Montagne Noire (France): biases for biodiversity documentation. Earth and Environmental Science Transactions of the Royal Society of Edinburgh, 93(4), pp.393-401.
3. Tortello, M.F., Vizcaïno, D. and Álvaro, J.J., 2006. Early Ordovician agnostoid trilobites from the southern Montagne Noire, France. Journal of Paleontology, 80(3), pp.477-495.
4. Serpagli, E., Ferretti, A., Vizcaino, D. and Álvaro, J.J., 2007. A new Early Ordovician conodont genus from the southern Montagne Noire, France. Palaeontology, 50(6), pp.1447-1457.
5. Courtessole, R., Pillet, J. and Vizcaïno, D., 1981. Nouvelles donnees sur la biostratigraphie de l'ordovicien inferieur de la Montagne Noire: révision des Taihungshaniidae, de Megistapis (Ekeraspis) et d'Asaphopsoides (Trilobites). Société des Etudes Scientifiques de l'Aude.
6. Courtessole, R., Pillet, J., Vizcaïno D., and Eschard, R., 1985. Étude biostratigraphique et sédimentologique des formations arénacées de l'Arenig du Saint-Chinianais oriental (Hérault), versant sud de la Montagne Noire (France méridionale). Mémoire de la Société d'Études Scientifiques de l'Aude, 99 p.
7. Lefebvre, B., Allaire, N., Guensburg, T.E., Hunter, A.W., Kouraïss, K., Martin, E.L., Nardin, E., Noailles, F., Pittet, B., Sumrall, C.D. and Zamora, S., 2016. Palaeoecological aspects of the diversification of echinoderms in the Lower Ordovician of central Anti-Atlas, Morocco. Palaeogeography, Palaeoclimatology, Palaeoecology, 460, pp.97-121.
8. Van Roy, P., Briggs, D.E. and Gaines, R.R., 2015. The Fezouata fossils of Morocco; an extraordinary record of marine life in the Early Ordovician. Journal of the Geological Society, 172(5), pp.541-549.
9. Gutiérrez-Marco, J.C. and Martin, E.L., 2016. Biostratigraphy and palaeoecology of Lower Ordovician graptolites from the Fezouata Shale (Moroccan Anti-Atlas). Palaeogeography, Palaeoclimatology, Palaeoecology, 460, pp.35-49.
10. Lehnert, O., Nowak, H., Sarmiento, G.N., Gutiérrez-Marco, J.C., Akodad, M. and Servais, T., 2016. Conodonts from the Lower Ordovician of Morocco—Contributions to age and faunal diversity of the Fezouata Lagerstätte and peri-Gondwana biogeography. Palaeogeography, Palaeoclimatology, Palaeoecology, 460, pp.50-61.
11. Nowak, H., Servais, T., Pittet, B., Vaucher, R., Akodad, M., Gaines, R.R. and Vandenbroucke, T.R., 2016. Palynomorphs of the Fezouata Shale (Lower Ordovician, Morocco): age and environmental constraints of the Fezouata Biota. Palaeogeography, Palaeoclimatology, Palaeoecology, 460, pp.62-74.
12. Lefebvre, B., Gutiérrez‐Marco, J.C., Lehnert, O., Martin, E.L., Nowak, H., Akodad, M., El Hariri, K. and Servais, T., 2018. Age calibration of the Lower Ordovician Fezouata Lagerstätte, Morocco. Lethaia, 51(2), pp.296-311.
13. Astini, R.A., Waisfeld, B.G., Toro, B.A. and Benedetto, J.L., 2004. El Paleozoico inferior y medio de la región de Los Colorados, borde occidental de la Cordillera Oriental (provincia de Jujuy). Revista de la Asociación Geológica Argentina, 59(2), pp.243-260.
14. Buatois, L.A., Zeballo, F.J., Albanesi, G.L., Ortega, G., Vaccari, N.E. and Mangano, M.G., 2006. Depositional environments and stratigraphy of the Upper Cambrian-Lower Ordovician Santa Rosita Formation at the Alfarcito area, Cordillera Oriental, Argentina: integration of biostratigraphic data within a sequence stratigraphic framework. Latin American journal of sedimentology and basin analysis, 13(1), pp.1-29.
15. Vaucher, R., Vaccari, N.E., Balseiro, D., Muñoz, D.F., Dillinger, A., Waisfeld, B.G. and Buatois, L.A., 2020. Tectonic controls on late Cambrian-Early Ordovician deposition in Cordillera oriental (Northwest Argentina). International Journal of Earth Sciences, 109, pp.1897-1920.
16. Waisfeld, B.G. and Vaccari, N.E., 2008. El género *Thysanopyge* (Trilobita, Ordovícico Temprano): especies y distribución en el noroeste argentino. Ameghiniana, 45(4), pp.753-774.
17. Toro, B.A., Arcerito, F.R.M., Muñoz, D.F., Waisfeld, B.G. and De La Puente, G.S., 2015. Graptolite-Trilobite Biostratigraphy in the Santa Victoria Area, Northwestern Argentina. A Key for Regional and Worldwide Correlation of the Lower Ordovician (Tremadocian—Floian). Ameghiniana, 52(5), pp.535-557.
18. Voldman, G.G., Albanesi, G.L., Ortega, G., Giuliano, M.E. and Monaldi, C.R., 2017. New conodont taxa and biozones from the Lower Ordovician of the Cordillera Oriental, NW Argentina. Geological Journal, 52(3), pp.394-414.
19. Serra, F., Balseiro, D. and Waisfeld, B.G., 2019. Diversity patterns in upper Cambrian to Lower Ordovician trilobite communities of north‐western Argentina. Palaeontology, 62(4), pp.677-695.
20. Saleh, F., Vaucher, R., Antcliffe, J.B., Daley, A.C., El Hariri, K., Kouraiss, K., Lefebvre, B., Martin, E.L., Perrillat, J.P., Sansjofre, P., Vidal, M., and Pittet, B., 2021. Insights into soft-part preservation from the Early Ordovician Fezouata Biota. Earth-Science Reviews, p.103464.
21. Mángano, M.G., Buatois, L.A. and Guinea, F.M., 2005. Ichnology of the Alfarcito Member (Santa Rosita Formation) of northwestern Argentina: animal-substrate interactions in a lower Paleozoic wave-dominated shallow sea. Ameghiniana, 42(4), pp.641-668.
22. Buatois, L.A. and Mángano, M.G., 2003. Sedimentary facies and depositional evolution of the Upper Cambrian–Lower Ordovician Santa Rosita formation in northwest Argentina. Journal of South American Earth Sciences, 16(5), pp.343-363.
23. Stegen, J.C., Lin, X., Fredrickson, J.K., Chen, X., Kennedy, D.W., Murray, C.J., Rockhold, M.L. and Konopka, A., 2013. Quantifying community assembly processes and identifying features that impose them. The ISME journal, 7(11), pp.2069-2079.
24. Clarke, K.R., 1993. Non‐parametric multivariate analyses of changes in community structure. Australian journal of ecology, 18(1), pp.117-143.
25. Gibert, C., 2020. DNCImper: assembly process identification based on SIMPER analysis. – R package ver. 0.0.1.0000, <https://github.com/Corentin-Gibert-Paleontology/DNCImper>.
26. Gibert, C., Shenbrot, G.I., Stanko, M., Khokhlova, I.S. and Krasnov, B.R., 2021. Dispersal-based versus niche-based processes as drivers of flea species composition on small mammalian hosts: inferences from species occurrences at large and small scales. Oecologia, 197(2), pp.471-484.
