# Supplementary material for: Contrasting Early Ordovician assembly patterns highlight the complex initial stages of the Ordovician Radiation
Source: Sci Rep. 2022 Mar 9;12:3852. doi: 10.1038/s41598-022-07822-z (PMC8907272; doi:10.1038/s41598-022-07822-z)
Supplement: Supplementary file 5 — Supplementary Information 5. [file 41598_2022_7822_MOESM5_ESM.html]

Script for DNCI analysis Saleh et al.


# Script for DNCI analyses Saleh et al.

#### Corentin Gibert & Farid Saleh

#### 08/06/2021

This is an R Markdown script for reproducing DNCI analysis from Saleh et al. with DNCImper package downloaded on GitHub: https://github.com/Corentin-Gibert-Paleontology/DNCImper.

### 1. Install the following libraries and sources.

library(factoextra)

library(FactoMineR)

library(vegan)

library(plyr)

#library(devtools)

#devtools::install\_github("Corentin-Gibert-Paleontology/DNCImper")

library(DNCImper)

If devtools can’t download the package, download manually the GitHub repository and install it with devtools:::install(DNCImper). Set your Working Directory in the folder containing the unzipped DNCImper repository.

You can find in https://github.com/Corentin-Gibert-Paleontology/PER-SIMPER-DNCI\_Tutorial a tutorial file for DNCI use named “Tutorial\_for\_R.R”.

This file will help you to use PerSIMPER, DNCI and other functions associated with Vilmi, Gibert et al. 2021 and Gibert & Escarguel 2019.

### 2. Tremadocian echinoderms in the Central Anti Atlas

The data required for this analysis can be found in the supplementary material 2 and is extracted into a .csv file “Echino CAA TR”.  
One should make sure that there are no empty columns in the .csv file.  
There are 4 clusters of Tremadocian echinoderms that differ in size, thus it is essential to use both overall and robustness functions.

EchinoCAA\_TR <- read.csv("Echino CAA TR.csv", h = T)

GroupEchinoCAA\_TR <- EchinoCAA\_TR[,1]

EchinoCAA\_TR <- EchinoCAA\_TR[,3:25]

Overall\_EchinoCAA\_TR <- DNCImper:::DNCI.ses\_overall(EchinoCAA\_TR, GroupEchinoCAA\_TR)

Overall\_EchinoCAA\_TR\_Robustesse <- DNCImper:::DNCI.ses\_overall\_symmetrized(EchinoCAA\_TR, GroupEchinoCAA\_TR, id = "Echino\_CAA\_TR", NbrReRun = 100)

### 3. Floian echinoderms in the Central Anti Atlas

The data required for this analysis can be found in the supplementary material 2 and is extracted into a .csv file “Echino CAA FL”.  
One should make sure that there are no empty columns in the .csv file.  
There are 2 clusters of Floian echinoderms that differ in size, thus only the robustness function should be used (the overall function is used only when having more than 2 clusters).

EchinoCAA\_FL <- read.csv("Echino CAA FL.csv", h = T)

GroupEchinoCAA\_FL <- EchinoCAA\_FL[,1]

EchinoCAA\_FL <- EchinoCAA\_FL[,3:17]

ByPairs\_EchinoCAA\_FL <-  DNCImper:::DNCI\_multigroup(EchinoCAA\_FL, GroupEchinoCAA\_FL, id = "Echino\_CAA\_FL", symmetrize = FALSE, count = FALSE)

ByPairs\_EchinoCAA\_FL\_Robustesse <- data.frame()

for(k in 1:100){

  ByPairs\_EchinoCAA\_FL\_Robustesse <- rbind(ByPairs\_EchinoCAA\_FL\_Robustesse,  DNCImper:::DNCI\_multigroup(EchinoCAA\_FL, GroupEchinoCAA\_FL, id = "Echino\_CAA\_FL", symmetrize = TRUE, count = FALSE))}

### 4. Tremadocian trilobites in the Central Anti-Atlas

The data required for this analysis can be found in the supplementary material 2 and is extracted into a .csv file “Trilo CAA TR”.  
One should make sure that there are no empty columns in the .csv file.  
There are 2 clusters of Tremadocian trilobites that differ in size, thus only the robustness function should be used (the overall function is used only when having more than 2 clusters).

TriloCAA\_TR <- read.csv("Trilo CAA TR.csv", h = T)

GroupTriloCAA\_TR <- TriloCAA\_TR[,1]

TriloCAA\_TR <- TriloCAA\_TR[,3:17]

ByPairs\_TriloCAA\_TR <-  DNCImper:::DNCI\_multigroup(ETriloCAA\_TR, GroupTriloCAA\_TR, id = “Trilo\_CAA\_TR”, symmetrize = FALSE, count = FALSE)

ByPairs\_TriloCAA\_TR\_Robustesse <- data.frame()

for(k in 1:100){

  ByPairs\_TriloCAA\_TR\_Robustesse <- rbind(ByPairs\_TriloCAA\_TR\_Robustesse,  DNCImper:::DNCI\_multigroup(TriloCAA\_TR, GroupTriloCAA\_TR, id = “Trilo\_CAA\_TR”, symmetrize = TRUE, count = FALSE))}

### 5. Floian trilobites in the Central Anti-Atlas

The data required for this analysis can be found in the supplementary material 2 and is extracted into a .csv file “Trilo CAA FL”.  
One should make sure that there are no empty columns in the .csv file.  
There are 2 clusters of Floian trilobites that differ in size, thus only the robustness function should be used (the overall function is used only when having more than 2 clusters).

TriloCAA\_FL <- read.csv("Trilo CAA FL.csv", h = T)

GroupTriloCAA\_FL <- TriloCAA\_FL[,1]

TriloCAA\_FL <- TriloCAA\_FL[,3:16]

ByPairs\_TriloCAA\_FL <-  DNCImper:::DNCI\_multigroup(ETriloCAA\_FL, GroupTriloCAA\_FL, id = “Trilo\_CAA\_FL”, symmetrize = FALSE, count = FALSE)

ByPairs\_TriloCAA\_FL\_Robustesse <- data.frame()

for(k in 1:100){

  ByPairs\_TriloCAA\_FL\_Robustesse <- rbind(ByPairs\_TriloCAA\_FL\_Robustesse,  DNCImper:::DNCI\_multigroup(TriloCAA\_FL, GroupTriloCAA\_FL, id = “Trilo\_CAA\_FL”, symmetrize = TRUE, count = FALSE))}

### 6. Tremadocian echinoderms in the Montagne Noire

The data required for this analysis can be found in the supplementary material 2 and is extracted into a .csv file “Echino MN TR”.  
One should make sure that there are no empty columns in the .csv file.  
There are 3 clusters of Tremadocian echinoderms that are not comparable in size.

EchinoMN\_TR <- read.csv("Echino MN TR.csv", h = T)

GroupEchinoMN\_TR <- EchinoMN\_TR[,1]

EchinoMN\_TR <- EchinoMN\_TR[,3:19]

Overall\_EchinoMN\_TR <-  DNCImper:::DNCI.ses\_overall(EchinoMN\_TR, GroupEchinoMN\_TR, count = FALSE)

Overall\_EchinoMN\_TR\_Robustesse <-  DNCImper:::DNCI.ses\_overall\_symmetrized(EchinoMN\_TR, GroupEchinoMN\_TR, id = "Echino\_MN\_TR", NbrReRun = 100)

### 7. Floian echinoderms in the Montagne Noire

The data required for this analysis can be found in the supplementary material 2 and is extracted into a .csv file “Echino MN FL”.  
One should make sure that there are no empty columns in the .csv file.  
There are 3 clusters of Floian echinoderms that are not comparable in size.

EchinoMN\_FL <- read.csv("Echino MN FL.csv", h = T)

GroupEchinoMN\_FL <- EchinoMN\_FL[,1]

EchinoMN\_FL <- EchinoMN\_FL[,3:17]

Overall\_EchinoMN\_FL <-  DNCImper:::DNCI.ses\_overall(EchinoMN\_FL, GroupEchinoMN\_FL, id = "Echino\_MN\_FL", count = FALSE)

Overall\_EchinoMN\_FL\_Robustesse <-  DNCImper:::DNCI.ses\_overall\_symmetrized(EchinoMN\_FL, GroupEchinoMN\_FL, id = "Echino\_MN\_FL", NbrReRun = 100)

### 8. Tremadocian trilobites in the Montagne Noire

The data required for this analysis can be found in the supplementary material 2 and is extracted into a .csv file “Trilo MN TR”.  
One should make sure that there are no empty columns in the .csv file.  
There are 3 clusters of Tremadocian trilobites that are not comparable in size.

TriloMN\_TR <- read.csv("Trilo MN TR.csv", h = T)

GroupTriloMN\_TR <- TriloMN\_TR[,1]

TriloMN\_TR <- TriloMN\_TR[,3:32]

Overall\_TriloMN\_TR <-  DNCImper:::DNCI.ses\_overall(TriloMN\_TR, GroupTriloMN\_TR, id = "Trilo\_MN\_TR")

Overall\_TriloMN\_TR\_Robustesse <-  DNCImper:::DNCI.ses\_overall\_symmetrized(TriloMN\_TR, GroupTriloMN\_TR, id = "Trilo\_MN\_TR", NbrReRun = 100)

### 9. Floian trilobites in the Montagne Noire

The data required for this analysis can be found in the supplementary material 2 and is extracted into a .csv file “Trilo MN FL”.  
One should make sure that there are no empty columns in the .csv file.  
There are 2 clusters of Floian trilobites that are not comparable in size.

TriloMN\_FL <- read.csv("Trilo MN FL.csv", h = T)

GroupTriloMN\_FL <- TriloMN\_FL[,1]

TriloMN\_FL <- TriloMN\_FL[,3:28]

ByPairs\_TriloMN\_FL <-  DNCImper:::DNCI\_multigroup(TriloMN\_FL, GroupTriloMN\_FL, id = "Trilo\_MN\_FL", symmetrize = FALSE, count = FALSE)

ByPairs\_TriloMN\_FL\_Robustesse <- data.frame()

for(k in 1:100){

  ByPairs\_TriloMN\_FL\_Robustesse <- rbind(ByPairs\_TriloMN\_FL\_Robustesse,  DNCImper:::DNCI\_multigroup(TriloMN\_FL, GroupTriloMN\_FL, id = "Trilo\_MN\_FL", symmetrize = TRUE, count = FALSE))}

### 10. Tremadocian trilobites in the Cordillera Oriental

The data required for this analysis can be found in the supplementary material 2 and is extracted into a .csv file “Trilo CO TR”.  
One should make sure that there are no empty columns in the .csv file.  
There are 4 clusters of Tremadocian trilobites that are not comparable in size.

TriloCO\_TR <- read.csv("Trilo CO TR.csv", h = T)

GroupTriloCO\_TR <- TriloCO\_TR[,1]

TriloCO\_TR <- TriloCO\_TR[,3:47]

Overall\_TriloCO\_TR <-  DNCImper:::DNCI.ses\_overall(TriloCO\_TR, GroupTriloCO\_TR)

Overall\_TriloCO\_TR\_Robustesse <-  DNCImper:::DNCI.ses\_overall\_symmetrized(TriloCO\_TR, GroupTriloCO\_TR, id = "Trilo\_CO\_TR", NbrReRun = 100)

### 11. Floian trilobites in the Cordillera Oriental

The data required for this analysis can be found in the supplementary material 2 and is extracted into a .csv file “Trilo CO FL”.  
One should make sure that there are no empty columns in the .csv file.  
There are 2 clusters of Floian trilobites that are not comparable in size.

TriloCO\_FL <- read.csv("Trilo CO FL.csv", h = T)

GroupTriloCO\_FL <- TriloCO\_FL[,1]

TriloCO\_FL <- TriloCO\_FL[,3:18]

ByPairs\_TriloCO\_FL <-  DNCImper:::DNCI\_multigroup(TriloCO\_FL, GroupTriloCO\_FL, id = "Trilo\_CO\_FL", symmetrize = FALSE, count = FALSE)

ByPairs\_TriloCO\_FL\_Robustesse <- data.frame()

for(k in 1:100){

  ByPairs\_TriloCO\_FL\_Robustesse <- rbind(ByPairs\_TriloCO\_FL\_Robustesse,  DNCImper:::DNCI\_multigroup(TriloCO\_FL, GroupTriloCO\_FL, id = "Trilo\_CO\_FL", symmetrize = TRUE, count = FALSE))}

## Results and extractions in .Rdata & .csv table

#In RDATA

All\_results <- list()

All\_results[[1]] <- Overall\_EchinoCAA\_TR

All\_results[[2]] <- Overall\_EchinoCAA\_TR\_Robustesse

All\_results[[3]] <- ByPairs\_EchinoCAA\_FL

All\_results[[4]] <- ByPairs\_EchinoCAA\_FL\_Robustesse

All\_results[[5]] <- ByPairs\_TriloCAA\_TR

All\_results[[6]] <- ByPairs\_TriloCAA\_FL

All\_results[[7]] <- Overall\_EchinoMN\_TR

All\_results[[8]] <- Overall\_EchinoMN\_TR\_Robustesse

All\_results[[9]] <- Overall\_EchinoMN\_FL

All\_results[[10]] <- Overall\_EchinoMN\_FL\_Robustesse

All\_results[[11]] <- Overall\_TriloMN\_TR

All\_results[[12]] <- Overall\_TriloMN\_TR\_Robustesse

All\_results[[13]] <- ByPairs\_TriloMN\_FL

All\_results[[14]] <- ByPairs\_TriloMN\_FL\_Robustesse

All\_results[[15]] <- Overall\_TriloCO\_TR

All\_results[[16]] <- Overall\_TriloCO\_TR\_Robustesse

All\_results[[17]] <- ByPairs\_TriloCO\_FL

All\_results[[18]] <- ByPairs\_TriloCO\_FL\_Robustesse

save(All\_results, file="All\_results.RDATA")

#In CSV table

TableExport <- data.frame()

TableExport <- rbind(TableExport, Overall\_EchinoCAA\_TR)

TableExport$id[1] <- "EchinoCAA\_TR"

TableExport <- rbind(TableExport, c("EchinoCAA\_TR\_Robustesse",1,2,mean(Overall\_EchinoCAA\_TR\_Robustesse$DELTA.dn),

                                    mean(Overall\_EchinoCAA\_TR\_Robustesse$CI.DELTA.dn),

                                    mean(Overall\_EchinoCAA\_TR\_Robustesse$S.DELTA.dn)))

TableExport <- rbind(TableExport, c("EchinoCAA\_FL",1,2,mean(ByPairs\_EchinoCAA\_FL$DNCI),

                                    mean(ByPairs\_EchinoCAA\_FL$CI.DNCI),

                                    mean(ByPairs\_EchinoCAA\_FL$S.DNCI)))

TableExport <- rbind(TableExport, c("EchinoCAA\_FL\_Robustesse",1,2,mean(ByPairs\_EchinoCAA\_FL\_Robustesse$DNCI),

                                    mean(ByPairs\_EchinoCAA\_FL\_Robustesse$CI.DNCI),

                                    mean(ByPairs\_EchinoCAA\_FL\_Robustesse$S.DNCI)))

TableExport <- rbind(TableExport, c("TriloCAA\_TR",1,2,mean(ByPairs\_TriloCAA\_TR$DNCI),

                                    mean(ByPairs\_TriloCAA\_TR$CI.DNCI),

                                    mean(ByPairs\_TriloCAA\_TR$S.DNCI)))

TableExport <- rbind(TableExport, c("TriloCAA\_FL",1,2,mean(ByPairs\_TriloCAA\_FL$DNCI),

                                    mean(ByPairs\_TriloCAA\_FL$CI.DNCI),

                                    mean(ByPairs\_TriloCAA\_FL$S.DNCI)))

TableExport <- rbind(TableExport, Overall\_EchinoMN\_TR)

TableExport$id[length(TableExport[,1])] <- "EchinoMN\_TR"

TableExport <- rbind(TableExport, c("EchinoMN\_TR\_Robustesse",1,2,mean(Overall\_EchinoMN\_TR\_Robustesse$DELTA.dn),

                                    mean(Overall\_EchinoMN\_TR\_Robustesse$CI.DELTA.dn),

                                    mean(Overall\_EchinoMN\_TR\_Robustesse$S.DELTA.dn)))

TableExport <- rbind(TableExport, Overall\_EchinoMN\_FL)

TableExport$id[length(TableExport[,1])] <- "EchinoMN\_FL"

TableExport <- rbind(TableExport, c("EchinoMN\_FL\_Robustesse",1,2,mean(Overall\_EchinoMN\_FL\_Robustesse$DELTA.dn),

                                    mean(Overall\_EchinoMN\_FL\_Robustesse$CI.DELTA.dn),

                                    mean(Overall\_EchinoMN\_FL\_Robustesse$S.DELTA.dn)))

TableExport <- rbind(TableExport, Overall\_TriloMN\_TR)

TableExport$id[length(TableExport[,1])] <- "TriloMN\_TR"

TableExport <- rbind(TableExport, c("TriloMN\_TR\_Robustesse",1,2,mean(Overall\_TriloMN\_TR\_Robustesse$DELTA.dn),

                                    mean(Overall\_TriloMN\_TR\_Robustesse$CI.DELTA.dn),

                                    mean(Overall\_TriloMN\_TR\_Robustesse$S.DELTA.dn)))

TableExport <- rbind(TableExport, c("TriloMN\_FL",1,2,mean(ByPairs\_TriloMN\_FL$DNCI),

                                    mean(ByPairs\_TriloMN\_FL$CI.DNCI),

                                    mean(ByPairs\_TriloMN\_FL$S.DNCI)))

TableExport <- rbind(TableExport, c("TriloMN\_FL\_Robustesse",1,2,mean(ByPairs\_TriloMN\_FL\_Robustesse$DNCI),

                                    mean(ByPairs\_TriloMN\_FL\_Robustesse$CI.DNCI),

                                    mean(ByPairs\_TriloMN\_FL\_Robustesse$S.DNCI)))

TableExport <- rbind(TableExport, Overall\_TriloCO\_TR)

TableExport$id[length(TableExport[,1])] <- "TriloCO\_TR"

TableExport <- rbind(TableExport, c("TriloCO\_TR\_Robustesse",1,2,mean(Overall\_TriloCO\_TR\_Robustesse$DELTA.dn),

                                    mean(Overall\_TriloCO\_TR\_Robustesse$CI.DELTA.dn),

                                    mean(Overall\_TriloCO\_TR\_Robustesse$S.DELTA.dn)))

TableExport <- rbind(TableExport, c("TriloCO\_FL",1,2,mean(ByPairs\_TriloCO\_FL$DNCI),

                                    mean(ByPairs\_TriloCO\_FL$CI.DNCI),

                                    mean(ByPairs\_TriloCO\_FL$S.DNCI)))

TableExport <- rbind(TableExport, c("TriloCO\_FL\_Robustesse",1,2,mean(ByPairs\_TriloCO\_FL\_Robustesse$DNCI),

                                    mean(ByPairs\_TriloCO\_FL\_Robustesse$CI.DNCI),

                                    mean(ByPairs\_TriloCO\_FL\_Robustesse$S.DNCI)))

write.csv(TableExport, "All\_results.csv")
