# Supplementary material for: Contrasting Early Ordovician assembly patterns highlight the complex initial stages of the Ordovician Radiation
Source: Sci Rep. 2022 Mar 9;12:3852. doi: 10.1038/s41598-022-07822-z (PMC8907272; doi:10.1038/s41598-022-07822-z)
Supplement: Supplementary file 6 — Supplementary Legends. [file 41598_2022_7822_MOESM6_ESM.docx]

**SM4 Caption**

Trilobite and echinoderm data files to be used to compute the dispersal niche continuum index.
